# The tumor suppressor NDRG2 recruits protein phosphatase 2A to suppress STAT5 phosphorylation in adult T-cell leukemia/lymphoma

**DOI:** 10.1016/j.jbc.2026.113080

**Published:** 2026-04-27

**Authors:** Tomonaga Ichikawa, Shingo Nakahata, Kazuya Shimoda, Takashi Murakami, Kazuhiro Morishita

**Affiliations:** 1 Department of Microbiology, Saitama Medical University, Saitama, Japan; 2 Division of Tumor and Cellular Biochemistry, Department of Medical Sciences, University of Miyazaki, Miyazaki, Japan; 3 Division of HTLV-1/ATL Carcinogenesis and Therapeutics, Joint Research Center for Human Retrovirus Infection, Kagoshima University, Kagoshima, Japan; 4 Faculty of Medicine, Division of Hematology, Diabetes, and Endocrinology, Department of Internal Medicine, University of Miyazaki, Miyazaki, Japan; 5 Faculty of Medicine, Division of Pediatrics, Developmental and Urological-Reproductive Medicine, University of Miyazaki, Miyazaki, Japan

**Keywords:** STAT5, NDRG2, PP2A, ATL, serine phosphorylation

## Abstract

Adult T-cell leukemia/lymphoma (ATL) is an aggressive T-cell malignancy with a poor prognosis that is caused by human T-cell leukemia virus type 1 infection. We previously demonstrated that N-myc downstream-regulated gene 2 (NDRG2) is significantly downregulated in ATL, resulting in aberrant activation of the signal transduction pathways through the dissociation of serine/threonine protein phosphatase 2A. To identify potential targets of NDRG2, we performed comprehensive mass spectrometry of differentially phosphorylated peptides in ATL cells with overexpression of NDRG2 using a TiO_2_-based enrichment method. Kyoto Encyclopedia of Genes and Genomes and gene ontology analysis revealed that the downregulated phosphopeptides correlated with signaling pathways, T-cell differentiation, and proliferation. Our results identified signal transducer and activator of transcription 5B as a novel NDRG2-regulated protein that is dephosphorylated at serine 193 and tyrosine 699. Although enforced expression of NDRG2 in ATL cell lines does not change the phosphorylation of Janus kinase 3, an upstream regulator of STAT5, phosphorylated STAT5 at tyrosine and serine is significantly suppressed by the direct binding to STAT5 with NDRG2 leading to the inhibition of STAT5 downstream gene expression. Furthermore, NDRG2 binds to STAT5B with alanine replacement of Y699 (Y699A), but only weakly associates with S193A, suggesting that NDRG2 is directly involved in serine phosphorylation through the recruitment of serine/threonine protein phosphatase 2A to STAT5. Because S193 A remarkably induces reduced phosphorylation of Y699 and subsequent transcriptional activity, the induction of serine phosphorylation through the loss of NDRG2 expression is dispensable for STAT5 tyrosine phosphorylation and activity. Since the loss of NDRG2 expression is essential factor to maintenance of ATL cells by STAT5 activity through phosphorylation of serine and tyrosine, targeting STAT5 becomes a feasible and effective strategy in NDRG2-deficient ATL.

Adult T-cell leukemia/lymphoma (ATL) is an aggressive cluster of differentiation (CD)4^+^ T lymphocyte malignancy with a poor prognosis that is associated with human T-cell leukemia virus type 1 (HTLV-1) infection. The oncogenic proteins HTLV-1 (Tax and HTLV-1 bZIP factor (HBZ)) are involved in ATL development and progression, with roles in regulating the accumulation of genetic and epigenetic abnormalities and posttranslational modifications of signaling molecules (1, 2). However, the precise mechanism of ATL pathogenesis is unknown, and diagnostic and therapeutic targets remain to be identified.

Recently, we reported a novel tumor suppressor gene, N-myc downstream-regulated gene 2 (NDRG2), that was significantly downregulated in ATL. Its downregulation is associated with the tumor incidence, progression, and metastasis in many types of cancers. NDRG2 expression inhibits the phosphorylation of many signaling molecules through the recruitment of serine/threonine protein phosphatase 2A (PP2A), negatively regulating signal transduction pathways. Downregulation of NDRG2 expression in ATL caused increased phosphorylation of phosphatase and tensin homolog deleted on chromosome 10 at S380, T382, and T383 through the disruption of PP2A recruitment, resulting in the constitutive activation of the phosphoinositide 3-kinase (PI3K)/protein kinase B (AKT) and nuclear factor kappa-light-chain-enhancer signaling pathways and leading to tumor development (3, 4, 5, 6). Identifying other phosphoproteins regulated by NDRG2 in ATL may lead to better understanding of the pathogenic mechanism of ATL and the development of therapeutic targets.

To explore the molecular mechanism of NDRG2 in ATL, we profiled the phosphorylated peptides that were modulated by NDRG2 using shotgun-based nano LC-MS with TiO_2_ phosphopeptide enrichment (7, 8). Among the results, we identified signal transducer and activator of transcription 5 (STAT5) as a novel NDRG2-regulated protein, which is highly phosphorylated in ATL. The constitutive activation of Janus kinase (JAK)/signaling transducer and activator of transcription (STAT) signaling pathway is involved in the progression and development of ATL- and other HTLV-1-infected diseases (9, 10, 11, 12).

The persistent phosphorylation of STAT family proteins is maintained by activated JAK family members through the involvement of autocrine IL-2/IL-2R and cytokine binding to the common cytokine chain (13, 14). Phosphorylated STAT molecules dimerize and enter the nucleus, leading to the upregulation of downstream target genes implicated in critical cellular processes such as cell proliferation, differentiation, and survival. STAT5A and STAT5B, which share more than 90% similarity in peptide sequence, are phosphorylated by phosphorylated JAK at tyrosine 694 and tyrosine 699, respectively, leading to increased transcriptional activity (15, 16). STAT5 activity is modulated by serine phosphorylation at S128, S726, S780 (STAT5A), S193, and S731 (STAT5B), which leads to protein stability and promotion of transcriptional activity (17, 18, 19, 20). However, the mechanism and maintenance of serine phosphorylation is not yet understood.

Our quantitative phosphoproteomics indicated that the phosphorylation of Y694 and Y699 (same sequence of phosphorylated peptide) and S193 (STAT5B) were significantly suppressed in ATL cells with enforced NDRG2 expression. Overexpression of NDRG2 in ATL cells led to the inhibition of STAT5 phosphorylation at tyrosine and serine without change of JAK3 phosphorylation, resulting in the reduction of STAT5 transcriptional activity and STAT5-target genes. STAT5B with alanine substitution at S193 (S193A), which eliminates phosphorylation at this site, showed decreased binding to NDRG2, indicating that NDRG2 interaction with STAT5 involves S193 phosphorylation. Moreover, the S193 A mutant showed reduced phosphorylation at C-terminal tyrosine residues and transcriptional activity. Together these results suggest that the presence of serine phosphorylation at STAT5 through the loss of NDRG2 expression induced the enhancement of tyrosine phosphorylation and the full activation of STAT5 followed by the onset and progression of ATL. Better understanding of the mechanisms that regulate phosphorylation and activation of STAT5 may lead to further elucidation of the pathways leading to ATL development. Inhibition of STAT5 inhibition may be a potential therapeutic approach for the diagnosis and therapy of ATL.

## Results

### Identification of STAT5A and STAT5B as potential targets of NDRG2

**Figure 1:**
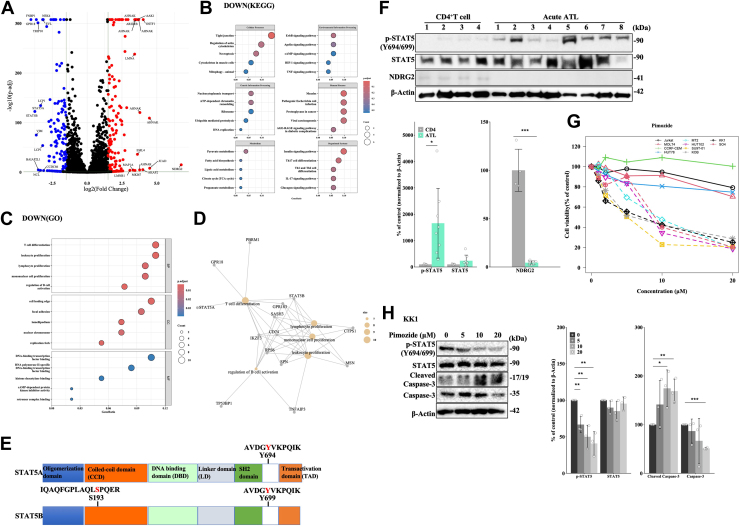
**Identification of STAT5A and STAT5B as potential targets of NDRG2.***A*, volcano plots of differentially phosphorylated peptides in the NDRG2 and control groups. Volcano plots of differentially phosphorylated peptides were plotted on the basis of the results of the DESeq2. The *x*-axis represents the log2 fold change, and the *y*-axis represents the -log10 (p-adj). *Red dots* indicate peptides with increased phosphorylation (log2(Fold Change) > 1.5 and p-adj  < 0.01), *blue dots* indicate peptides with decreased phosphorylation (log2(fold change) < −1.5 and p-adj  < 0.01), and *gray dots* indicate peptides that were not differentially phosphorylated. The phosphorylated peptides with |log2(fold change)| > 3 are annotated with their gene names. *B*, top five KEGG pathways of differentially downregulated phosphopeptides. The *x*-axis indicates the percentage of peptides over the total peptides in a given pathway, and the *y*-axis indicates KEGG pathways. *Circle sizes* represent the number of peptides in each function, and *bubble colors* correspond to *p* values. *C*, *top* 5 GO pathways of differentially downregulated phosphopeptides. The *x*-axis indicates the percentage of the number of peptides present in this GO term over the total number of peptides in this category, and the *y*-axis indicates GO terms of three categories (BP: biology process, CC: cell component, MF: molecular function). *Circle sizes* represent the number of peptides in each function, and *bubble colors* correspond to *p* values. *D*, Cnetplot of GO enrichment analysis. Cnetplot displays the *Top* 5 GO BP terms and protein name of related peptides. *Circle sizes* represent the number of peptides in each pathway. *E*, schematic representation of the structure of STAT5A and STAT5B and the location of candidate phosphorylation sites. *F*, Western blot analysis of phosphorylated (p)-STAT5 (Y694/699), STAT5, and NDRG2 in CD4+ T lymphocytes from healthy volunteers (n = 4) and primary ATL cells from patients with acute-type ATL (n = 8). Bar graphs show quantification of the relative band intensity normalized to β-actin. Data are shown as mean and SD; ∗*p* < 0.05, ∗∗∗*p* < 0.001 *versus* CD4 group. *G*, cell viability and IC50 were determined using Cell Counting Kit-8 assays in non-ATL and ATL-related cell lines after treatment with 0 to 20 μM pimozide for 120 h. *H*, KK1 cells were treated with the indicated doses of pimozide for 48 h, followed by immunoblot analysis. The results are representative of three independent experiments. Bar graphs show the quantification of the relative band intensity normalized to β-actin with or without pimozide treatment. Data are shown as mean and SD (n = 3); **∗***p* < 0.05, ∗∗*p* < 0.01, ∗∗∗*p* < 0.001 *versus* 0 h control. STAT, signal transducer and activator of transcription; NDRG2, N-myc downstream-regulated gene 2; KEGG, Kyoto Encyclopedia of Genes and Genomes; GO, gene ontology; BP, biological process; ATL, adult T-cell leukemia/lymphoma; CD, cluster of differentiation.

We previously identified the NDRG2 tumor suppressor as a PP2A recruiter that negatively regulates signaling pathways in ATL by dephosphorylating important signaling molecules (3). To comprehensively analyze the proteins targeted by NDRG2, we introduced a Flag-tagged NDRG2 expression vector or empty vector (Mock) into the KOB ATL cell line, and the differentially phosphorylated peptides were profiled by quantitative phosphoproteomics between control (parental and Mock) and NDRG2 group. Our analysis identified 284 differentially phosphorylated peptides (153 with upregulation of phosphorylation, 131 with downregulation of phosphorylation), as shown in the volcano plot (|log2(fold change)| >1.5, p-adj < 0.01) in Figure 1*A*. The top peptide groups dephosphorylated included formin binding protein 1, never in mitosis A-related kinase 4, G-coupled receptor 18, thyroid hormone receptor interactor 10, and Enah/Vasp-like. A chromosomal translocation t (9, 11) (q34;q23) involving MLL (KMT2A) and formin binding protein 1 has been reported in isolated cases of acute leukemia (21). We also detected peptides of STAT5, which plays an important role in ATL pathogenesis through the JAK/STAT signal transduction pathway, with decreased phosphorylation in the NDRG2 group compared with the control (Fig. 1*A*).

To determine the physiological functions of the proteins corresponding to the differentially phosphorylated peptides, we performed Kyoto Encyclopedia of Genes and Genomes (KEGG) and gene ontology (GO) enrichment analysis. KEGG enrichment analysis identified many pathways related with tight junction, several signal pathways, and T-cell differentiation (Fig. 1*B*). GO analysis revealed terms related to T cells, such as T-cell differentiation, leukocyte proliferation, and lymphocyte proliferation in biological processes (Fig. 1*C*). Cnetplot revealed the distribution and relationship of the downregulated phosphopeptides, and STAT5B was shown to play a central role in each pathway (Fig. 1*D*). The upregulated phosphopeptides in the NDRG2 group were associated with chromosome functions (Fig. S1, *A* and *B*).

We examined the phosphorylated peptide sequences of STAT5A and STAT5B and identified putative dephosphorylation sites at tyrosine (Y) 694 in STAT5A (AVDG(pY)VKPQIK) and serine (S) 193 and Y699 in STAT5B (IQAQFGPLAQL(pS)PQER and AVDG(pY)VKPQIK, respectively) (Fig. 1*E* and Table S1). Although the expression levels of STAT5 protein were similar in patient-derived primary ATL cells and healthy CD4^+^T cells, the NDRG2 protein expression in primary ATL cells was remarkably lower than that in CD4^+^T cells and, the phosphorylation of STAT5A at Y694 and STAT5B at Y699 was significantly increased in most primary ATL samples (Fig. 1*F*). Furthermore, the dose-dependent transfection of EGFP-NDRG2 into 293T cells with Flag-STAT5A/B revealed a marked reduction of STAT5 phosphorylation at Y694 or Y699 in a dose-dependent manner (Fig. S1*C*), suggesting that the phosphorylation of STAT5 may be inversely proportional to NDRG2 expression.

We next evaluated the effects of JAK/STAT inhibitors ruxolitinib and pimozide in three T-cell acute lymphoblastic leukemia (T-ALL) cell lines (Jurkat, MOLT4, and CCRF-CEM), one cutaneous T cell lymphoma (CTCL) cell line (HUT78), two HTLV-1-infected cell lines (MT2, HUT102), and four ATL cell lines (SU9T-01, KOB, KK1, and SO4) after 120 h. Ruxolitinib is a potent selective and orally bioavailable JAK inhibitor that suppresses phosphorylated STAT5 and cell proliferation (13, 22). Ruxolitinib exhibited antiproliferative activity in all ATL-associated cell lines; in contrast, non-ATL (T-ALL and CTCL) cell lines did not respond (Fig. S1*D* and Table S2). The neuroleptic drug pimozide is an inhibitor of STAT5 (23, 24). All ATL-related cell lines were more sensitive to pimozide than T-ALL and CTCL cell lines (Fig. 1*G* and Table S2). Pimozide treatment of the ATL cell line significantly suppressed STAT5 phosphorylation in a dose-dependent manner, accompanied by the promotion of cell apoptosis as detected by cleaved caspase-3 (Fig. 1*H*). These findings indicate STAT5A/B may be targets of NDRG2, suggesting that tyrosine and serine phosphorylation of STAT5A/B were regulated by NDRG2/PP2A *via* direct and/or indirect mechanisms.

### NDRG2 suppresses STAT5 activity by dephosphorylation in ATL cells

**Figure 2:**
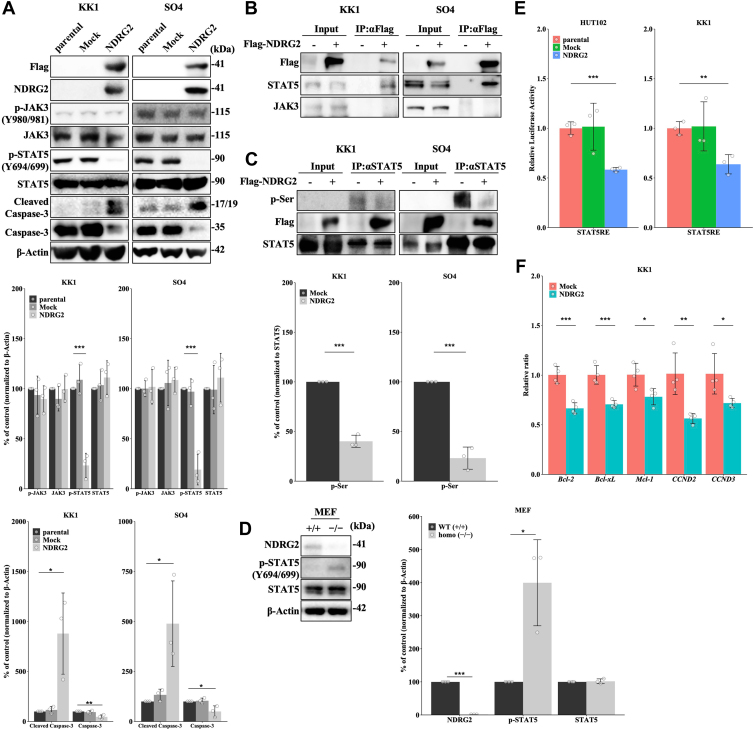
**NDRG2 suppresses STAT5 activity by dephosphorylation in ATL cells.***A*, immunoblot analysis of KK1 and SO4 cells (parental, Mock, and NDRG2). Bar graphs show the quantification of relative band intensity normalized to β-actin. Data are shown as mean and SD (n = 3); ∗*p* < 0.05, ∗∗*p* < 0.01, ∗∗∗*p* < 0.001 *versus* parental. *B*, cell lysates from KK1 and SO4 cells (Mock and NDRG2) were precipitated using anti-Flag antibody, and the precipitated proteins were immunoblotted. *C*, cell lysates from the KK1 and SO4 cells (Mock and NDRG2) were precipitated using anti-STAT5 antibody, and the precipitated proteins were immunoblotted. Bar graphs show the quantification of the relative band intensity normalized to immunoprecipitated STAT5. Data are shown as mean and SD (n = 3); ∗∗∗*p* < 0.001 *versus* Mock. *D*, cell lysate form NDRG2 WT (+/+) and homo (−/−) MEF was investigated using specific antibodies in immunoblots. Bar graphs show the quantification of the relative band intensity normalized to β-actin. The mean and SD are shown (n = 3); ∗*p* < 0.05, ∗∗∗*p* < 0.001 *versus* WT (+/+). *E*, HUT102 and KK1 cells were transfected with pSTAT5RE-Luc and pRL-TK plasmids and subjected to luciferase reporter assays. Data are shown as mean and SD (n = 3); ∗∗*p* < 0.01, ∗∗∗*p* < 0.001 *versus* parental. *F*, quantitative PCR analysis of STAT5-related genes in KK1 cells transfected as indicated. Data are shown as mean and SD (n = 4); ∗*p* < 0.05, ∗∗*p* < 0.01, ∗∗∗*p* < 0.001 *versus* Mock. NDRG2, N-myc downstream-regulated gene 2; STAT, signal transducer and activator of transcription; MEF, mouse embryonic fibroblast.

To explore whether NDRG2 modulates the JAK/STAT signaling pathway in ATL cell lines, we investigated STAT5 phosphorylation (at Y694/699) in ATL cell lines with NDRG2 overexpression. The JAK kinase phosphorylates and activates STAT5, and activation of JAK by phosphorylation is a key event triggering STAT signaling; therefore, we evaluated JAK phosphorylation. Notably, despite the undetectable expression of NDRG2 in parental and Mock ATL cell lines, STAT5 phosphorylation at Y694/699 was significantly suppressed by NDRG2 overexpression and induction of cell apoptosis was observed, confirming STAT5 as a target of NDRG2. However, the phosphorylation status of JAK3 was unchanged (Fig. 2*A* and S2*A*).

Coimmunoprecipitation assays showed that Flag-NDRG2 interacted with STAT5, but not JAK3 in ATL cell lines (Fig. 2*B* and S2*B*). NDRG2 expression led to a decrease of STAT5 serine phosphorylation in ATL cell lines (Fig. 2*C* and S2*C*). In addition, the inhibition of NDRG2 expression in T-ALL cells (Jurkat and MOLT4) resulted in the increase of STAT5 phosphorylation at Y694 or Y699 without detectable changes in JAK3 phosphorylation compared with controls (Fig. S2*D*). Therefore, we analyzed the phosphorylation of STAT5 in mouse embryonic fibroblast (MEF) from *NDRG2*-deficient (−/−) and WT (+/+) mice. *NDRG2* homo (−/−) MEF exhibited significantly decreased expression of NDRG2 and increased phosphorylation of STAT5 at Y694 or Y699 compared with WT (+/+) MEF (Fig. 2*D*). We next evaluated STAT5 transcriptional activity using a luciferase reporter vector containing STAT5 response element (STAT5RE) and the results showed that STAT5 transcriptional activity was reduced in ATL cell lines with NDRG2 overexpression (Fig. 2*E*). Moreover, the levels of STAT5-regulated target genes were significantly decreased in ATL cell lines with NDRG2 overexpression compared with controls (Fig. 2*F* and S2*E*). These results indicate that NDRG2 might interact with and dephosphorylates STAT5, independent of the regulation of JAK activity, leading to the suppression of STAT5 transcriptional activity.

### The regulation of tyrosine phosphorylation in STAT5 through serine phosphorylation by NDRG2 in ATL

**Figure 3:**
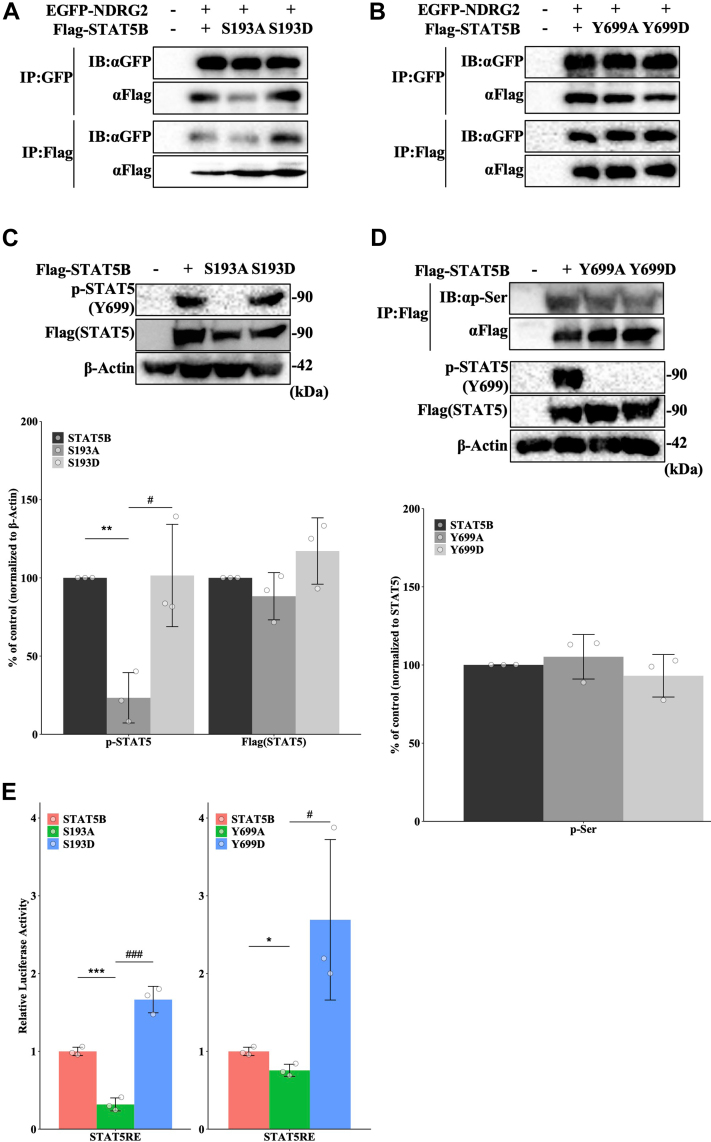
**The regulation of tyrosine phosphorylation in STAT5 through serine phosphorylation by NDRG2 in ATL.***A*, 293T cells were cotransfected with EGFP-NDRG2 and Flag-STAT5B mutants (WT, S193A, and S193D), and immunoprecipitates were immunoblotted with anti-GFP or anti-Flag antibodies. *B*, 293T cells were cotransfected with EGFP-NDRG2 and Flag-STAT5B mutants (WT, Y699A, and Y699D), and immunoprecipitates were immunoblotted with anti-GFP or anti-Flag antibodies. *C*, 293T cells were transfected with Flag-STAT5B mutants (WT, S193A, and S193D), and whole lysates were subjected to immunoblot analysis. Bar graphs show the quantification of relative band intensity normalized to β-actin. Data are shown as mean and SD (n = 3); ∗∗*p* < 0.01 *versus* STAT5B, and #*p* < 0.05 *versus* S193A. *D*, 293T cells were transfected with Flag-STAT5B mutants (WT, Y699A, and Y699D); cell lysates were precipitated using anti-Flag antibody, and the precipitated proteins were immunoblotted. Bar graphs show the quantification of the relative band intensity normalized to immunoprecipitated STAT5. Data are shown as mean and SD (n = 3). *E*, 293T cells were transfected with Flag-STAT5B mutant, pSTAT5RE-Luc, and pRL-TK plasmids and subjected to luciferase reporter assays. Data are shown as mean and SD (n = 3); ∗*p* < 0.05, ∗∗∗*p* < 0.001 *versus* STAT5B; ###*p* < 0.001 *versus* S193A; #*p* < 0.05 *versus* Y699A. STAT, signal transducer and activator of transcription; NDRG2, N-myc downstream-regulated gene 2.

To investigate in more detail how NDRG2 regulates STAT5B, we engineered a STAT5B expression vector with an alanine substitution at 193 or 699 (S193A, Y699A), to ablate phosphorylation at this site, or aspartic acid substitution at 193 or 699 (S193D, Y699D), to mimic high phosphorylation. Coimmunoprecipitation experiments showed that EGFP-NDRG2 coprecipitated with WT STAT5B and the S193D mutant, but exhibited weak association with the S193A mutant (Fig. 3*A*). The binding affinity of NDRG2 to the Y699A and Y699D mutants was preserved (Fig. 3*B*). We then looked at the impact of STAT5B phosphorylation on other phosphorylation sites. Although the STAT5B WT and S193D proteins showed phosphorylation at Y699, the STAT5B S193A mutant showed significantly reduced phosphorylation at Y699 compared with the WT. Serine phosphorylation of STAT5B was unaffected in the Y699A and Y699D mutant proteins (Fig. 3*C* and *D*).

Transcriptional activity of the phosphorylation-deficient S193A and Y699A mutants was reduced compared with WT STAT5B, whereas the phosphomimetic S193D and Y699D mutants exhibited transcriptional activity (Fig. 3*E*). We performed similar analyses of STAT5A mutants (S193A, S193D, Y694A, and Y694D). We observed no significant difference in binding activity of NDRG2 to STAT5A and STAT5B, and the results obtained with STAT5A mutants mirrored those of the STAT5B mutants (Fig. S3*A–E*). Collectively, these findings suggest that NDRG2 interacts with or near the S193 phosphorylation site in STAT5B and phosphorylation of this site plays a role in STAT5B transcriptional activity.

### Regulation of STAT5 through dephosphorylation by NDRG2/PP2A

**Figure 4:**
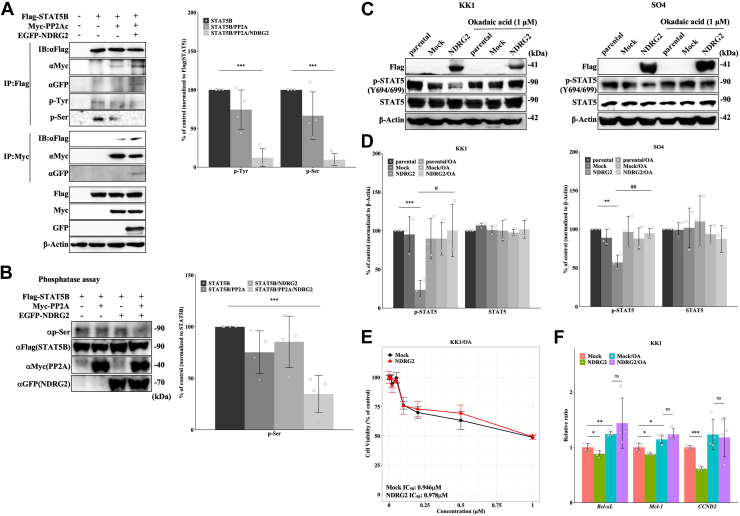
**Regulation of STAT5 through dephosphorylation by NDRG2/PP2A.***A*, cell lysates of 293T cells transfected with Flag-STAT5B, Myc-PP2A, and EGFP-NDRG2 were immunoprecipitated with anti-Flag or anti-Myc antibodies, and immunoprecipitants were detected by Western blotting. Bar graphs show the quantification of the relative band intensity (p-Tyr and p-Ser) normalized to immunoprecipitated Flag (STAT5). Data are shown as mean and SD (n = 4); ∗∗∗*p* < 0.001 *versus* STAT5B. *B*, the phosphatase activity of immunoprecipitates for 293T cells transfected with Myc-PP2A and/or EGFP-NDRG2 with each specific antibody was measured using an *in vitro* phosphatase assay with Flag-STAT5B as the substrate. The results are representative of four independent experiments. Bar graphs show the quantification of the relative band intensity normalized to STAT5B. Data are shown as mean and SD (n = 4); ∗∗∗*p* < 0.001 *versus* STAT5B. *C*, KK1 and SO4 cells (parental, Mock, and NDRG2) were pretreated with or without okadaic acid (1 μM) for 24 h, followed by Western blot analysis. *D*, bar graphs show the quantification of relative band intensity normalized to β-actin. Data are shown as mean and SD (n = 3); ∗∗*p* < 0.01, ∗∗∗*p* < 0.001 *versus* parental; #*p* < 0.05, ##*p* < 0.01 *versus* NDRG2. *E*, cell viability and IC50 were determined using Cell Counting Kit-8 assays in KK1 (Mock and NDRG2) cells after treatment with 0 to 1 μM OA for 48 h. Data are shown as mean and SD (n = 4). *F*, quantitative PCR analysis of STAT5-related genes in KK1 (Mock and NDRG2) cells treated with or without OA (1 μM) for 24 h. Data are shown as mean and SD (n = 4); ∗*p* < 0.05, ∗∗*p* < 0.01, ∗∗∗*p* < 0.001 *versus* Mock; ns (not significant) *versus* Mock/OA. STAT, signal transducer and activator of transcription; NDRG2, N-myc downstream-regulated gene 2; PP2A, serine/threonine protein phosphatase 2A.

We next examined the involvement of the serine/threonine phosphatase PP2A in STAT5 phosphorylation. Flag-STAT5, Myc-PP2A, and/or EGFP-NDRG2 were introduced into 293T cells and protein interactions were assessed by coimmunoprecipitation assays. PP2A was detected in STAT5 protein complexes, and there was a tendency toward the suppression of phosphorylated STAT5 by PP2A. Furthermore, the formation of the PP2A/STAT5 complex was enhanced in the presence of NDRG2, leading to a significant decrease in both tyrosine and serine phosphorylation of STAT5 (Fig. 4*A* and S4*A*). To investigate whether the presence of NDRG2 regulates STAT5B serine phosphorylation through the recruitment of PP2A, the phosphatase activity of Myc-PP2A and/or EGFP-NDRG2 transiently expressed in 293T cells purified by immunoprecipitation with each tagged antibody was measured using an *in vitro* phosphatase assay with Flag-STAT5B as a substrate. The phosphorylation of STAT5B at serine did not significantly change *via* the immunoprecipitated PP2A or NDRG2; however, the combination of immunoprecipitated PP2A and NDRG2 markedly decreased STAT5B serine phosphorylation (Fig. 4*B*). These finding suggest that NDRG2 and PP2A could not independently regulate STAT5 dephosphorylation, and the presence of NDRG2 recruited PP2A to STAT5, thereby inducing the dephosphorylation of STAT5. We next investigated whether a PP2A inhibitor, okadaic acid (OA), influences tyrosine phosphorylation of STAT5 in ATL cells with NDRG2 expression. OA treatment significantly abolished NDRG2-induced dephosphorylation of STAT5 at Y694/699 in ATL cell lines (Fig. 4, *C* and *D* and S4, *B* and *C*). OA induced ubiquitin-mediated degradation of Bcl-2, Bcl-xL, CDK4, and Cyclin D2 protein *via* phosphorylation through inhibition of PP2A activity, leading to apoptosis in ATL and HTLV-1 related cells (25). On the other hand, inhibition of PP2A activity by OA increased IL-8 mRNA through the activation of signal transduction pathways by unrestrained phosphorylation in cancer cells, and maintained high level of Cyclin D transcription (26, 27). To investigate whether treatment with OA was involved in cell viability of ATL cells through NDRG2 expression, we compared with IC_50_ value between Mock and NDRG2-KK1 cells treated with OA. Although growth rates of ATL cells were significantly suppressed by NDRG2 overexpression compared with Mock (Fig. S4*D*), the introduction of NDRG2 in ATL cells showed no significant sensitivity to OA treatment compared with Mock, with IC_50_ value of 0.946 μM at 48 h for Mock *versus* NDRG2 of 0.978 μM in OA (Fig. 4*D*). Although the treatment with OA increased or maintained the expression of STAT5-regulated target genes in Mock ATL cells, gene expression induced by OA in NDRG2 ATL cells remained at same level as OA-treated Mock ATL cells without the decline of transcription (Fig. 4*F*). We indicated that PP2A and NDRG2 function in a cooperative manner, and thus the inhibition of PP2A activity restores the effects mediated by NDRG2 overexpression. We strongly suggest that PP2A plays a pivotal role in the effects of NDRG2 on modulating STAT5 dephosphorylation. We speculate that in ATL cells, the absence of NDRG2 expression leads to augmented STAT5 serine phosphorylation through the disruption of PP2A. This impairment results in the constitutive activation of the JAK/STAT signaling pathway *via* sustained tyrosine phosphorylation and may contribute to ATL development.

## Discussion

Our previous investigation demonstrated that the tumor suppressor NDRG2 functions to inhibit PI3K/AKT and nuclear factor kappa-light-chain-enhancer signaling pathways through the dephosphorylation of phosphorylation of phosphatase and tensin homolog deleted on chromosome 10 *via* the recruitment of the PP2A phosphatase. Downregulation of NDRG2 expression in ATL was linked to the aberrant activation of signaling pathways and promoted tumor development (3, 28).

The present study reveals that NDRG2 is critically involved in modulating the phosphorylation status of key cellular regulatory molecules that may be involved in the regulation of T-cell function, signaling pathways, and nuclear function. Our results identified STAT5A and STAT5B as targets of NDRG2. We demonstrated that NDRG2-mediated dephosphorylation of STAT5 at serine and tyrosine is intricately linked to the inactivation of STAT5 transcriptional activity, suggesting that loss of NDRG2 expression in ATL may lead to elevated phosphorylation of STAT5 at S193 through the dissociation of PP2A, leading to the enhancement of STAT5 signaling and tumor development.

The constitutive activation of the JAK/STAT signaling pathway is involved in proliferation and survival of malignancies including ATL (10, 11, 12). STAT5 is phosphorylated and activated by JAK phosphorylation, resulting in the enhancement of transcriptional activity and promotion of tumorigenesis (29, 30, 31). Cytokine-induced phosphorylation at S128, S726, and S780 in STAT5A and S193 and S731 in STAT5B has been reported in many types of cancers (17, 18, 19, 20). However, the regulatory mechanisms governing STAT5 function in ATL have not been fully elucidated. Phosphorylation of STAT5B at S193 was detected in hematopoietic malignancies including ATL, leading to enhanced transcriptional activity. Other studies showed that S193 phosphorylation of STAT5B induced by IL-2 stimulation is negatively regulated by mechanistic target of rapamycin (mTOR) inhibitors (such as rapamycin) and positively regulated by PP2A inhibition with OA (17). Here, we identified S193 in STAT5B as a potential target of NDRG2 using quantitative phosphoproteomics and demonstrated that NDRG2 overexpression significantly induced the dephosphorylation of S193, Y694 (STAT5A), and Y699 (STAT5B) in ATL cells.

STAT5 activity regulated by phosphorylation is modulated by receptor tyrosine kinases, cytokine receptors, and nonreceptor tyrosine kinases (15, 16). mTOR, downstream of PI3K/AKT, directly induces STAT5 phosphorylation at S193 (17). We speculate that NDRG2 may regulate S193 dephosphorylation of STAT5 by inactivating mTOR through the inhibition of AKT phosphorylation. Our results showed that phosphorylation of JAK3, an upstream regulator of STAT5, was unaffected by NDRG2 in ATL cells. Moreover, our data show a role for PP2A in the effects of NDRG2 on STAT5. Thus, we speculate that NDRG2 binds to STAT5B, followed by dephosphorylation at S193 *via* the recruitment of PP2A, resulting in the suppression of C-terminal phosphorylation (Fig. S5*A*).

The phosphorylation and activity of JAK/STAT signaling molecules are increased in hematologic cancers, and this pathway has attracted attention as a therapeutic target. Several JAK inhibitors have been used for treatment of rheumatoid arthritis, myelofibrosis, and tumors (32, 33). Ruxolitinib inhibits the phosphorylation of JAK and the tyrosine phosphorylation of STAT3/5, resulting in the suppression of oncogenic target genes and cell proliferation in many types of tumors (13, 22). Pimozide is an antipsychotic drug approved by the US Food and Drug Administration. It was reported to show effective anticancer potential through the inhibition of signaling pathways followed by the induction of apoptosis and autophagy (23, 24). Pimozide treatment significantly suppressed cell proliferation and STAT5 phosphorylation in ATL cells (23, 24, 34). The development of new drugs for cancer treatment is time consuming and costly. In contrast, drug repositioning, the process of finding new therapeutic uses for existing drugs, is an effective strategy for accelerating drug development and reducing investment risk (34).

In conclusion, we identified STAT5 as a novel NDRG2-regulated protein and showed that phosphorylation of S193 and Y694/699 in STAT5 is constitutively induced in NDRG2 low expression ATL cells, leading to the increase of oncogenic gene expression and cell proliferation. Loss of NDRG2 in ATL may cause the retention of S193 phosphorylation through the dissociation of PP2A, followed by the enhancement of tyrosine phosphorylation and transcriptional activity, indicating that tyrosine and serine phosphorylation of STAT5 is necessary for its activity and tumorigenesis in ATL without NDRG2 expression (Fig. S5*B*). Therefore, inhibitors targeting STAT5 may be novel therapeutic agents for many malignancies, including ATL.

## Experimental procedures

### Reagents

Ruxolitinib was purchased from Selleck Chemicals. Pimozide was obtained from Sigma-Aldrich. OA was purchased from Cayman Chemical. The cell proliferation/cell toxicity kit (Cell Counting Kit-8) was purchased from DOJINDO. Most antibodies were purchased from companies listed in Table S3.

### Shotgun-nano LC-MS

Cells were fixed with 100% methanol and digested in buffer (1 M urea and 50 mM NH_4_HCO_3_) containing MS-grade trypsin (Promega) at 37 °C for 20 h with mixing. The reaction was stopped by acidification with 0.8% formic acid (final concentration), and peptide solution was centrifuged at 15,000 × *g* for 5 min. An equal volume of ethyl acetate was added to the supernatant, and the resulting lower aqueous phase was dried. Phosphopeptide enrichment was performed by Titansphere Phos-TiO kit (GL Sciences) according to the manufacturer’s instructions. The resultant peptide solution was resuspended in 0.1% formic acid at a concentration of 500 ng/μl prior to analysis by LC-MS. MS was performed on the Q Exactive orbitrap MS (Thermo Fisher Scientific). The resulting phosphopeptide solution was analyzed using the UltiMate3000 RSLCnano (Thermo Fisher Scientific) system, and loaded on an EASY-Spray column (25 cm × 75 μm ID, PepMap C18, 2 μm; Thermo Fisher Scientific) with a 70 min gradient at flow rate of 300 μl/min. Solvent A was water/0.1% formic acid, and solvent B was acetonitrile/0.1% formic acid; peptides were eluted using a gradient from 1% to 35% solvent B. The measurement method of peptides has been described (35).

### Quantitative phosphoproteomics

The count data of control (parental, n = 4, Mock, n = 4) and NDRG2 (n = 8) in ATL cell line KOB were compared using the R package DESeq2, and differentially phosphorylated peptides (padj < 0.01, log2 fold-change; positive or negative) were identified. In R, the value of the smallest floating-point was 2.225074e-308; thus, 0 was replaced with 2.225074e-308 (36). We identified upregulated peptides (log2(fold change) > 1.5 and p-adj < 0.01) and downregulated peptides (log2(fold change) < - 1.5 and p-adj < 0.01) in the NDRG2 group compared with the control group. The R package ‘clusterProfiller’ was used to perform KEGG and GO enrichment analysis using the following thresholds: a *p*-value cut-off of 1 and a q-value cut-off of 0.5 (37, 38). Enrichment analysis was performed for the upregulated and downregulated peptides. Cnetplot was used to determine the complex associations among the significantly phosphorylated peptides that exhibit potential biological complexity.

### Patient samples

This study was performed in accordance with the Declaration of Helsinki and the Japanese Ethical Guidelines for Medical and Health Research Involving Human Subjects and for Human Genomic/Genetic Analysis Research. Blood samples were obtained with informed consent under the approval of the Institutional Review Board of the Faculty of Medicine, University of Miyazaki (Approval Code: 972, Approval Data: January 17, 2001), and the Ethical Committee of Saitama Medical University (Approval Code: 2023-013, Approval Data: July 11, 2023). Peripheral blood mononuclear cells were isolated using Lymphoprep (Axis-Shield). ATL cells were collected from patients at hospital admission before the start of chemotherapy. CD4^+^ T lymphocytes were isolated from blood samples of healthy volunteers by negative selection using an AutoMACS with a CD4^+^ T-cell isolation kit (Miltenyi Biotec). The ATL cells were maintained in AIM-V medium (Thermo Fisher Scientific) supplemented with 20% FBS, 10 μM 2-mercaptoethanol (Thermo Fisher Scientific), and 10 ng/ml recombinant human IL-2 (PeproTech).

### Cell lines

Jurkat, MOLT4, and CCR-CEM are HTLV-1-negative human T-ALL cell lines. HUT78 is CTCL cell line. MT2 and HUT102 are human T cell lines transformed by HTLV-1 infection. SU9T-01 is IL-2-independent ATL cell lines. KOB, KK1, and SO4 are interleukin-2 (IL-2)-dependent ATL cell lines. Jurkat and MOLT4 cells were obtained from Fujisaki Cell Center, Hayashibara Biochemical Laboratories. CCR-CEM, HUT78 and Human embryonic kidney cell lines HEK293T (293T) are obtained from RIKEN Bioresource Center (BRC). MT2 and HUT102 cells were kindly donated by Dr H. Iha (Oita University). SU9T-01 was gifted by Dr N. Arima (Kagoshima University). KOB, KK1 and SO4 were kind gifts from Dr Y. Yamada (Nagasaki University). The procedure used to isolate MEF has been previously described (3). IL-2-dependent ATL cells were maintained in RPMI 1640 medium (Nacalai Tesque) supplemented with 10% FBS and 10 ng/ml recombinant human IL-2 in a humidified atmosphere of 5% CO_2_ at 37 °C. HTLV-1-negative, HTLV-1-infected, and IL-2-independent ATL cell lines were maintained in the same medium, without IL-2. 293T, and MEF from WT (+/+) and *NDRG2*-deficient (−/−) mice were cultured in Dulbecco’s modified Eagle’s medium (Nacalai Tesque) supplemented with 10% FBS. *Mycoplasma* testing was performed using MycoAlert *Mycoplasma* Detection Kit (LONZA).

### Plasmids

The Flag-tagged NDRG2 (Flag-NDRG2), short hairpin RNA (shRNA) expression vector targeting luciferase (shluc) as a control, and NDRG2 (shNDRG2) have been described previously (3). Myc-tagged PP2A (Myc-PP2A) was kind gifts from Dr H. Shima (Miyagi Cancer Center Research Institute, Japan). The enhanced green fluorescent protein (EGFP)-tagged NDRG2 (EGFP-NDRG2) generated using Flag-NDRG2 constructs as templates and subcloned into the pEGFP-C1 (Clontech, Mountain View, CA). The full-length complementary DNA (cDNA) sequence of STAT5A (NM_003152.4) and STAT5B (NM_012448.4) subcloned into the p3xFlag-myc-CMV26 expression vector (Sigma-Aldrich) (Flag-STAT5A and STAT5B). To generate the vectors expressing substitution mutants of STAT5A and STAT5B, PCR-based mutagenesis was performed to induce mutations in the coding sequences using mutagenic primers (Table S4) followed by DpnI treatment. Transient transfections were performed using polyethylenimine hydrochloride (PEI-MAX; Polysciences) and Amaxa Cell Line Nucleofector Kit V (LONZA) according to the manufacturer’s protocol.

### Western blot

Cells were harvested for protein extraction by homogenization in NP-40 lysis buffer (50 mM Tris-HCl, pH 8.0, 150 mM NaCl, 5 mM EDTA, and 1% NP-40) supplemented with a proteinase inhibitor cocktail (Sigma-Aldrich) and phosphatase inhibitor tablet (PhosSTOP). The lysate was centrifuged at 15,000 × *g* (maximum) for 10 min at 4 °C and the supernatant was collected. The supernatant protein concentration was measured using a bovine serum albumin (BSA) standard. Equal amounts of protein samples were boiled at 95 °C for 10 min in 1 × SDS sample buffer (62.5 mM Tris-HCl, pH 6.8, 2% SDS, 25% glycerol, 5% 2-mercaptoethanol, and 0.01% bromophenol blue), separated by SDS-PAGE, and transferred to a polyvinylidene difluoride membrane (Immobilon-P; Millipore). The membranes were blocked in TBS (10 mM Tris-HCl, pH 7.4, and 100 mM NaCl)-Tween (0.1%) (TBST) with 1% BSA or Blocking One (Nacalai Tesque) prior to incubation with primary antibodies (1:1000) in TBST-BSA or Can Get Signal Solution 1 (TOYOBO) overnight at 4 °C. After washing three times with TBST, the membranes were incubated with horseradish peroxidase-conjugated secondary antibodies diluted in TBST-BSA or Can Get Signal Solution 2 (TOYOBO) at room temperature for 1 h. Bands were detected using a EZWestLumi plus (ATTO) and an LAS-3000 imager (Fujifilm). The band intensities were quantified using ImageJ software (National Institutes of Health).

### Immunoprecipitation

Lysates were incubated with 1 μg of the indicated antibodies or normal IgG at 4 °C overnight with constant rotation, followed by incubation with Protein G Sepharose 4 Fast Flow (GE Healthcare) for 2 h. Next, the immunoprecipitates were washed three times with PBS and the bound proteins were denatured in SDS sample buffer. Each sample was subjected to Western blot analysis.

### cDNA synthesis and quantitative PCR (qPCR)

Total RNA was isolated from the cells using NucleoSpin RNA Plus (TaKaRa Bio). Next, cDNA was synthesized from 500 ng of RNA using an PrimeScript RT Master Mix (TaKaRa Bio). qPCR was performed using a LightCycler96 system (Roche), Brilliant III Ultra-Fast SYBR Green QPCR Master Mix (Agilent Technologies), and THUNDERBIRD Next SYBR qPCR Mix (TOYOBO). The expression levels of the target genes were normalized to β-actin mRNA levels. Table S5 lists all primer sequences.

### Cell proliferation assay

Cells were seeded in 96-well plates at a density of 3 × 10^3^ cells/well and incubated for the indicated time periods. Viable cells were counted using a methylthiazolyl tetrazolium assay with Cell Counting Kit-8. The 50% inhibitory concentration (IC_50_) values were calculated using the following formula: IC_50_ = 10^LOG(*A*/*B*) × (50 −^ ^*C*)/(D - C) + LOG(*B*)^, where *A* is the concentration of the upper side of 50% absorbance, *B* is the concentration of the lower side of 50% absorbance, *C* is the rate of absorbance reduction at a concentration of *B*, and *D* is the rate of absorbance reduction at a concentration of *A*.

### Luciferase assay

Cells were transfected with luciferase reporter vector containing STAT5 response element (pGL4.52[luc2P/STAT5RE], Promega, pSTAT5RE-Luc) and pGL4.74 Renilla luciferase plasmid (pGL-4.74[hRluc/TK], Promega) as a transfection control vector using transfection reagents. The transcriptional activity was measured using Dual-Luciferase Reporter Assay System (Promega) according to the manufacturer's instructions with Varioskan LUX Multimode Microplate Reader (Thermo Fisher Scientific).

### *In vitro* phosphatase assay

A modified *in vitro* phosphatase assay was performed, as previously described (39, 40). Briefly, extracts from 293T cells expressing tagged molecules (Flag, Myc, and EGFP) were immunoprecipitated using each tagged antibody. After immunoprecipitation, the beads were washed twice in a low-stringency buffer containing 20 mM Hepes (pH 8), 50 mM NaCl, 0.1 mM EDTA, and 2.5 mM MgCl_2_ and once in phosphatase assay buffer containing 100 mM Tris-HCl pH 8, 10 mM DTT (Nacalai Tesque). The phosphatase reactions were performed in 50 μl of phosphatase assay buffer and incubated for 40 min at 37 °C. The beads were washed three times with phosphatase assay buffer and the reactions were stopped by the addition of SDS sample buffer, followed by boiling. The reaction products were subjected to immunoblotting.

### Statistical analysis

No statistical methods were used to predetermine sample size. Sample size was defined according to our previous experience. Each experiment was independently performed at least three times. Results were not blinded for analysis. Data, bars, and markers in the figures represent mean ± SD. We used the two-tailed Student’s *t* test for comparisons within each parameter, and ANOVA and Dunnett’s multiple comparisons test for multiple comparisons or to compare several different treatments with a single control. Statistical analyses were performed using Prism software 9 (GraphPad) and R package. Differences were considered statistically significant at *p* < 0.05, 0.01, and 0.001.

## Data availability

All raw data generated in this article are available upon request from the corresponding author.

## Supporting information

This article contains supporting information.

## Conflict of interest

T. M. has financial interests in Denka Co., Ltd. S. N., K. S., K. M., and T. I. declare no conflicts of interest with the contents of this article.

## Supporting information

Supporting Information

**Abbreviations**
AKT, protein kinase B
ATL, adult T-cell leukemia/lymphoma
BSA, bovine serum albumin
CD, cluster of differentiation
cDNA, complementary DNA
CTCL, cutaneous T cell lymphoma
GO, gene ontology
HBZ, HTLV-1 bZIP factor
HTLV-1, Human T-cell leukemia virus type 1
JAK, Janus kinase
KEGG, Kyoto Encyclopedia of Genes and Genomes
MEF, mouse embryonic fibroblast
mTOR, mechanistic target of rapamycin
NDRG2, N-myc downstream-regulated gene 2
NF-κB, nuclear factor kappa-light-chain-enhancer
OA, okadoic acid
PI3K, phosphoinositide 3-kinase
PP2A, serine/threonine protein phosphatase 2A
qPCR, quantitative PCR
STAT, signal transducer and activator of transcription
T-ALL, T cell acute lymphoblastic leukemia
