# Supplementary material for: The tumor suppressor NDRG2 recruits protein phosphatase 2A to suppress STAT5 phosphorylation in adult T-cell leukemia/lymphoma
Source: J Biol Chem. 2026 Apr 27;302(6):113080. doi: 10.1016/j.jbc.2026.113080 (PMC13226920; doi:10.1016/j.jbc.2026.113080)
Supplement: Supporting Information [file mmc1.pdf]

Table S1. Identification of candidate dephosphorylation of STAT5 by NDRG2

Gene Symbol	Accession	Annotated Sequence	Modification	Modifications in Master Proteins	Log2FC	P-adj
STAT5A	P42229	[K].AVDGYVKPQIK.[Q]	1xPhospho [Y5(100)]	[Y694(100)]	-3.749	3.13E-123
STAT5B	P51692	[R].IQAQFGPLAQLSPQER.[L]	1xPhospho [S12(100)]	[S193(100)]	-0.887	2.89E-08
		[K].AVDGYVKPQIK.[Q]	1xPhospho [Y5(100)]	[Y699(100)]	-3.749	3.13E-123

Table S2. Inhibitory effect of various JAK/STAT inhibitors on cell proliferation of non-ATL and ATL-related cells at 120h.

Cell lines	Ruxolitinib	Pimozide
Jurkat	>10	>100
MOLT4	>10	30.35
CCRF-CEM	>10	>100
HUT78	>10	99.05
MT2	0.27	9.25
HUT102	0.5	8.03
SU9T-01	1.44	5.06
KOB	0.23	5.7
KK1	0.74	6.66
SO4	0.22	8.71

The numbers represent IC50 (μM).

Table S3. The list of the antibodies used in this manuscript.

Antibody	Manufacturer	Catalog no	Type
p-STAT5(Tyr694)(D47E7)	Cell Signaling	#4322	Rabbit monoclonal
STAT5(D206Y)	Cell Signaling	#94205	Rabbit monoclonal
p-JAK3(Tyr980/981)(D44E3)	Cell Signaling	#5031	Rabbit monoclonal
JAK3(D1H3)	Cell Signaling	#8827	Rabbit monoclonal
Cleaved Caspase-3(Asp175)(5A1E)	Cell Signaling	#9664	Rabbit monoclonal
Caspase-3(D3R6Y)	Cell Signaling	#14220	Rabbit monoclonal
GFP (D5.1)	Cell Signaling	#2956	Rabbit monoclonal
DYKDDDK Tag(D6W5B)(Flag)	Cell Signaling	#14793	Rabbit monoclonal
Myc-Tag (9B11)	Cell Signaling	#2276	Mouse monoclonal
NDRG2(E-20)	SANTA CRUZ	sc-19468	Goat polyclonal
GFP(B-2)	SANTA CRUZ	sc-9996	Mouse monoclonal
p-Ser (16B4)	SANTA CRUZ	sc-81514	Mouse monoclonal
p-Tyr (PY20)	SANTA CRUZ	sc-508	Mouse monoclonal
Flag(M2)	Sigma-Aldrich	F3165	Mouse monoclonal
β-actin(AC-74)	Sigma-Aldrich	A5316	Mouse monoclonal
Polyclonal Rabbit anti-Mouse IgG/HPR	Dako	P0260	
Polyclonal Swine anti-Rabbit IgG/HPR	Dako	P0399	
Polyclonal Rabbit anti-Goat IgG/HPR	Dako	P0449	

Table S4. Primer list for the amino acid replacement of STAT5A and STAT5B.

Mutation site	Sequence (5' to 3')
STAT5A S193	TTGCCCAGCTGGCCCAGCTG**<u>AGC</u>**CCCCAGGAGCGTCT GAGCCGGGAGACG (1199-1248)
STAT5A S193A (serine 193-alanine)	TTGCCCAGCTGGCCCAGCTG**<u>GCC</u>**CCCCAGGAGCGTCT GAGCCGGGAGACG (S193A)
STAT5A S193D (serine 193-aspartic acid)	TTGCCCAGCTGGCCCAGCTG**<u>GAC</u>**CCCCAGGAGCGTCT GAGCCGGGAGACG (S193D)
STAT5A Y694	TGGCTAAAGCTGTTGATGGA**<u>TAT</u>**GTGAAACCACAGA TCAAGCAAGTGGTC (2702-2751)
STAT5A Y694A (tyrosine 694-alanine)	TGGCTAAAGCTGTTGATGGA**<u>GCT</u>**GTGAAACCACAGA TCAAGCAAGTGGTC (Y694A)
STAT5A Y694D (tyrosine 694-aspartic acid)	TGGCTAAAGCTGTTGATGGA**<u>GAT</u>**GTGAAACCACAGA TCAAGCAAGTGGTC (Y694D)
STAT5B S193	TTGGCCCGCTGGCCCAGCTG**<u>AGC</u>**CCCCAGGAGCGTCT GAGCCGGGAGACG (711-760)
STAT5B S193A (serine 193-alanine)	TTGGCCCGCTGGCCCAGCTG**<u>GCC</u>**CCCCAGGAGCGTCT GAGCCGGGAGACG (S193A)
STAT5B S193D (serine 193-aspartic acid)	TTGGCCCGCTGGCCCAGCTG**<u>GAC</u>**CCCCAGGAGCGTCT GAGCCGGGAGACG (S193D)
STAT5B Y699	CTGCTAAAGCTGTTGATGGA**<u>TAC</u>**GTGAAGCCACAGA TCAAGCAAGTGGTC (2229-2278)
STAT5B Y699A (tyrosine 699-alanine)	CTGCTAAAGCTGTTGATGGA**<u>GCC</u>**GTGAAGCCACAGA TCAAGCAAGTGGTC (Y699A)
STAT5B Y699D (tyrosine 699-aspartic acid)	CTGCTAAAGCTGTTGATGGA**<u>GAC</u>**GTGAAGCCACAGA TCAAGCAAGTGGTC (Y699D)

Table S5. Primer list for real-time qPCR.

Gene		Sequence (5' to 3')
Bcl-2	F	CGGTGGGGTCATGTGTGTG
	R	CGGTTCAGGTACTCAGTCATCC
Bcl-xL	F	GAGCTGGTGGTTGACTTTCTC
	R	TCCATCTCCGATTCAGTCCCT
Mcl-1	F	TGCTTCGGAAACTGGACATCA
	R	TAGCCACAAAGGCACCAAAAG
CCND2	F	GAGAAGCTGTCTCTGATCCGCA
	R	CTTCCAGTTGCGATCATCGACG
CCND3	F	AGATCAAGCCGCACATGCGGAA
	R	ACGCAAGACAGGTAGCGATCCA
β-actin	F	GACAGGATGCAGAAGGAGAT
	R	TGATCCACATCTGCTGGAAGGT

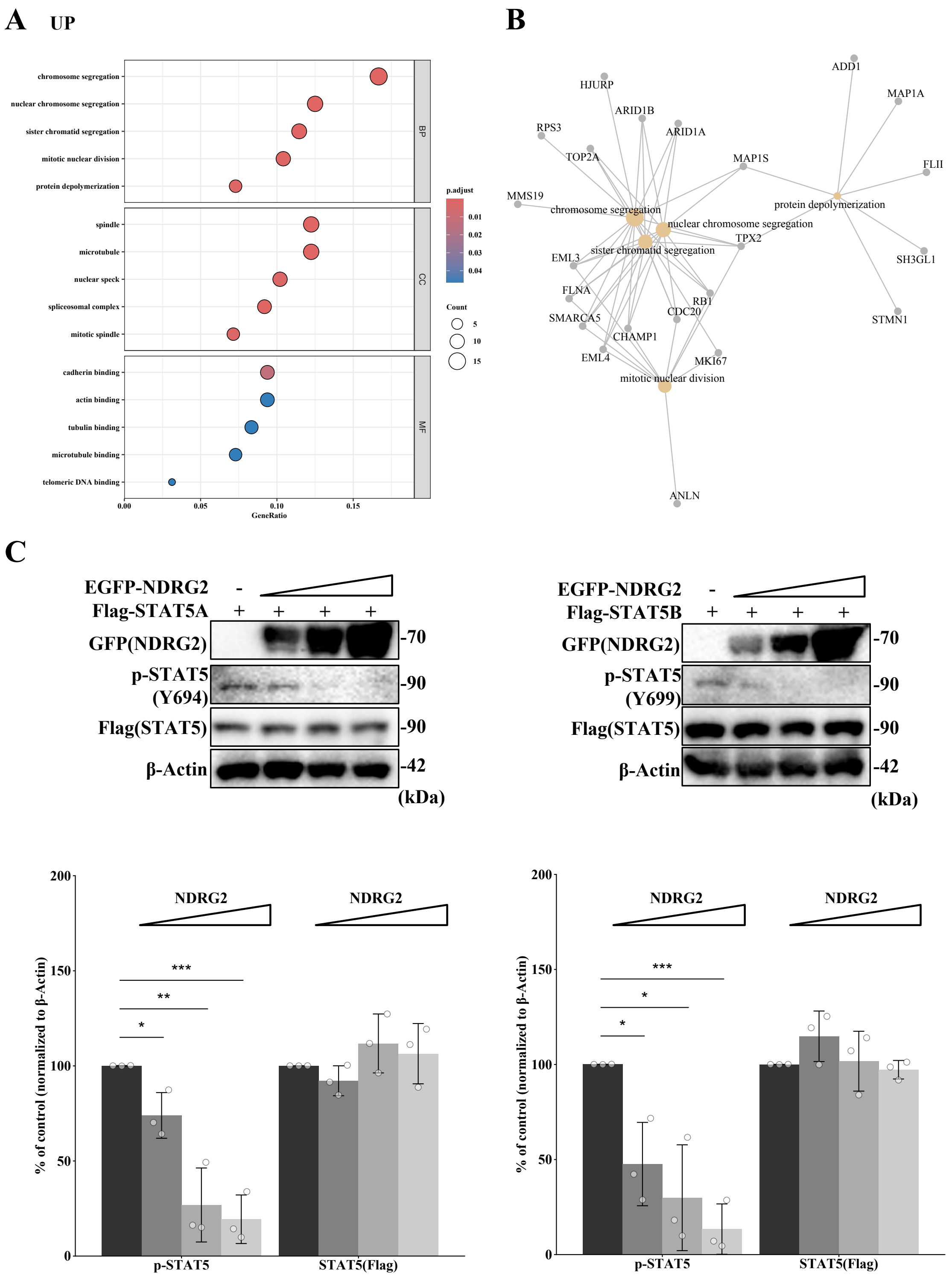
A
UP
chromosome segregation
nuclear chromosome segregation
sister chromatid segregation
mitotic nuclear division
protein depolymerization
spindle
microtubule
nuclear speck
spliceosomal complex
mitotic spindle
cadherin binding
actin binding
tubulin binding
microtubule binding
telomeric DNA binding
BP
CC
MF
0.00
0.05
0.10
0.15
GeneRatio
p.adjust
0.01
0.02
0.03
0.04
Count
5
10
15
B
ADD1
HJURP
MAP1A
ARID1B
RPS3
ARID1A
TOP2A
MAP1S
FLII
MMS19
protein depolymerization
chromosome segregation
nuclear chromosome segregation
TPX2
sister chromatid segregation
EML3
SH3GL1
FLNA
RB1
CDC20
SMARCA5
STMN1
CHAMP1
EML4
MKI67
mitotic nuclear division
ANLN
size
8
10
12
14
16
C
EGFP-NDRG2
Flag-STAT5A
GFP(NDRG2)
p-STAT5
(Y694)
Flag(STAT5)
β-Actin
-70
-90
-90
-42
(kDa)
EGFP-NDRG2
Flag-STAT5B
GFP(NDRG2)
p-STAT5
(Y699)
Flag(STAT5)
β-Actin
-70
-90
-90
-42
(kDa)
% of control (normalized to β-Actin)
NDRG2
NDRG2
***
**
*
p-STAT5
STAT5(Flag)
% of control (normalized to β-Actin)
NDRG2
NDRG2
***
*
*
p-STAT5
STAT5(Flag)

D

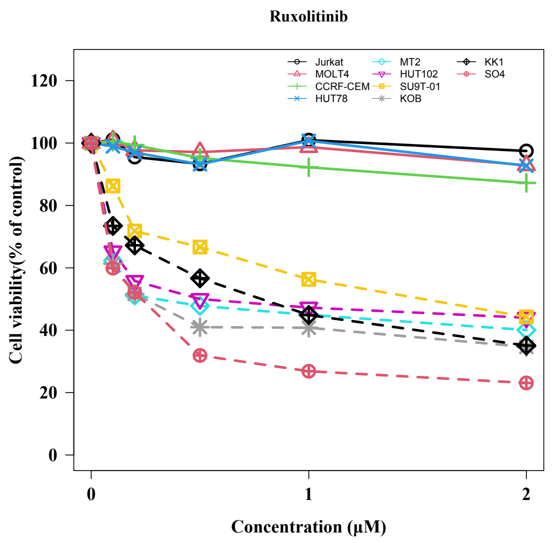

Figure S1.

A, Top five GO pathways of differentially upregulated phosphopeptides. The x-axis indicates the percentage of the number of peptides present in this GO term over the total number of peptides in this category, and the y-axis indicates GO terms of three categories (BP: biology process, CC: cell component, MF: molecular function). Circle sizes represent the number of peptides in each function, and bubble colors correspond to p values. B, Cnetplot of GO enrichment analysis. Cnetplot displays the Top 5 GO: BP terms and protein name of related peptides. Circle sizes represent the number of peptides in each pathway. C, 293T cells were co-transfected with increasing amounts of EGFP-NDRG2 and same amount of Flag-STAT5A/B, and subjected to western blot analysis with the indicated antibodies. Bar graphs show the quantification of the relative band intensity normalized to β-actin. Data are shown as mean and SD (n = 3); *$p < 0.05$, **$p < 0.01$, ***$p < 0.001$ versus non-transfected with NDRG2. D, Cell viability and IC_{50} were determined using Cell Counting Kit-8 assay in non-ATL and ATL-related cell lines after treatment with 0–2 μM Ruxolitinib for 120 h.

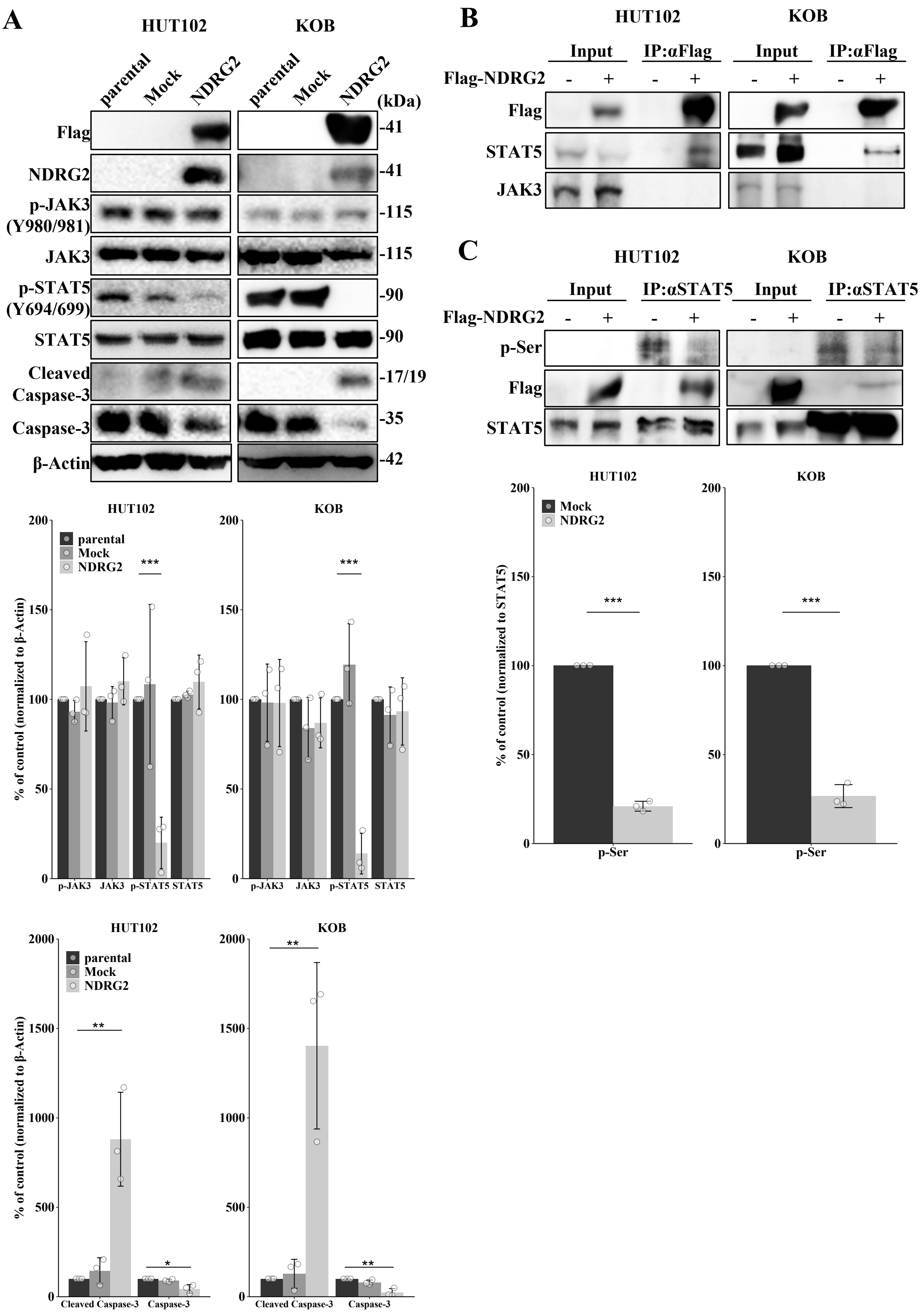
A
HUT102
KOB
parental
Mock
NDRG2
(kDa)
Flag
NDRG2
p-JAK3
(Y980/981)
JAK3
p-STAT5
(Y694/699)
STAT5
Cleaved
Caspase-3
Caspase-3
β-Actin
-41
-41
-115
-115
-90
-90
-17/19
-35
-42
B
HUT102
KOB
Input
IP:αFlag
Flag-NDRG2
Flag
STAT5
JAK3
C
HUT102
KOB
Input
IP:αSTAT5
Flag-NDRG2
p-Ser
Flag
STAT5
% of control (normalized to β-Actin)
p-JAK3
JAK3
p-STAT5
STAT5
***
Cleaved Caspase-3
Caspase-3
**
*
% of control (normalized to STAT5)
p-Ser

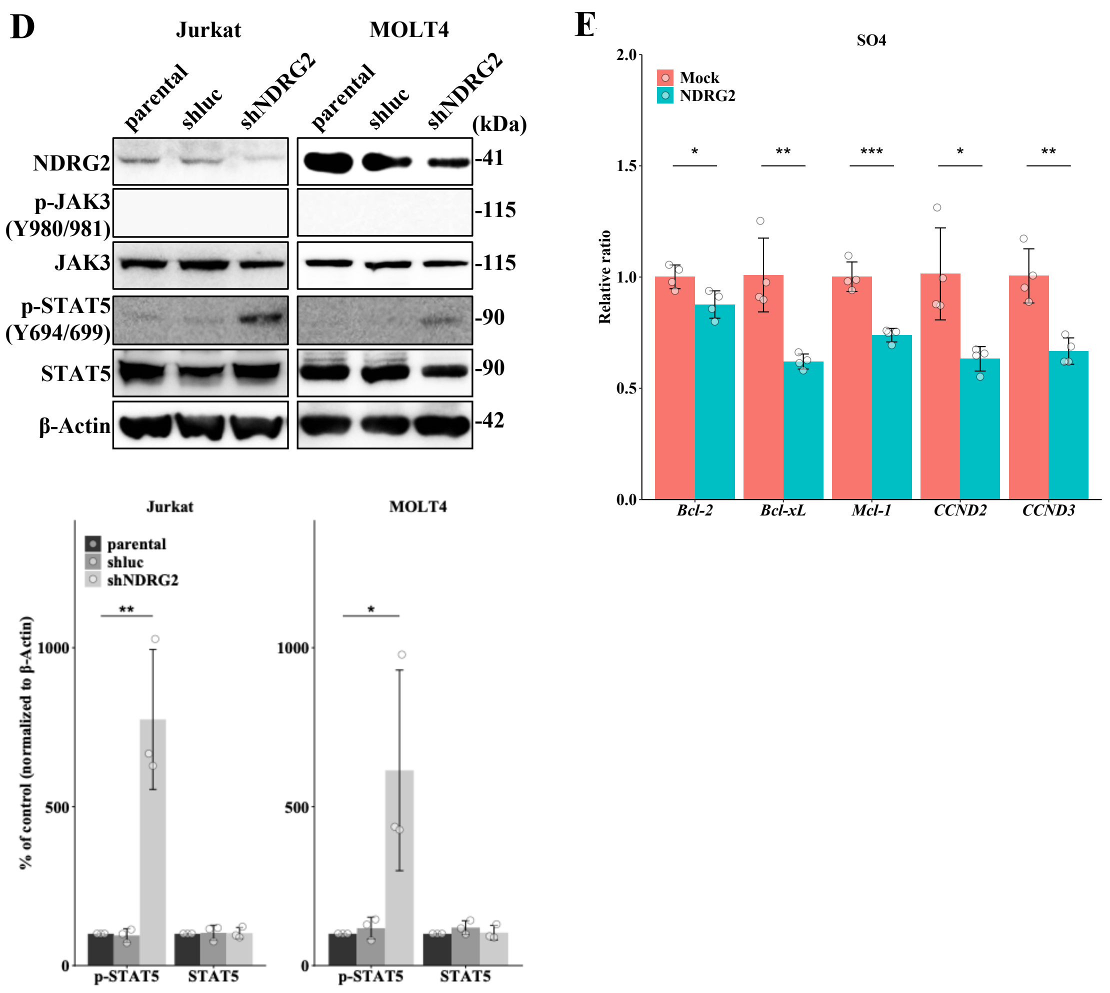

Figure S2.

A, Cell lysate from HUT102 and KOB cells (parental, Mock, and NDRG2) was investigated using antibodies specific to each immunoblot. Bar graphs show the quantification of relative band intensity normalized to β-actin. The mean and SD are shown (n = 3); *$p < 0.05$, **$p < 0.01$, ***$p < 0.001$ versus parental. B, Cell lysates from the HUT102 and KOB cells (Mock and NDRG2) were precipitated using anti-Flag antibody, and the precipitated proteins were immunoblotted with each specific antibody. C, Cell lysates from the HUT102 and KOB cells (Mock and NDRG2) were precipitated using anti-STAT5 antibody, and the precipitated proteins were immunoblotted

with each specific antibody. Bar graphs show the quantification of the relative band intensity normalized to immunoprecipitated STAT5. The mean and SD are shown (n = 3); ***$p < 0.001$ versus Mock. D, Cell lysate from Jurkat and MOLT4 cells (parental, shluc, and shNDRG2) was investigated using antibodies specific to each immunoblot. Bar graphs show the quantification of relative band intensity normalized to β-actin. The mean and SD are shown (n = 3); *$p < 0.05$, **$p < 0.01$ versus parental. E, Quantitative PCR analysis of STAT5-related genes in SO4 cells (Mock and NDRG2). The mean and SD are shown (n = 4); *$p < 0.05$, **$p < 0.01$, ***$p < 0.001$ versus Mock.

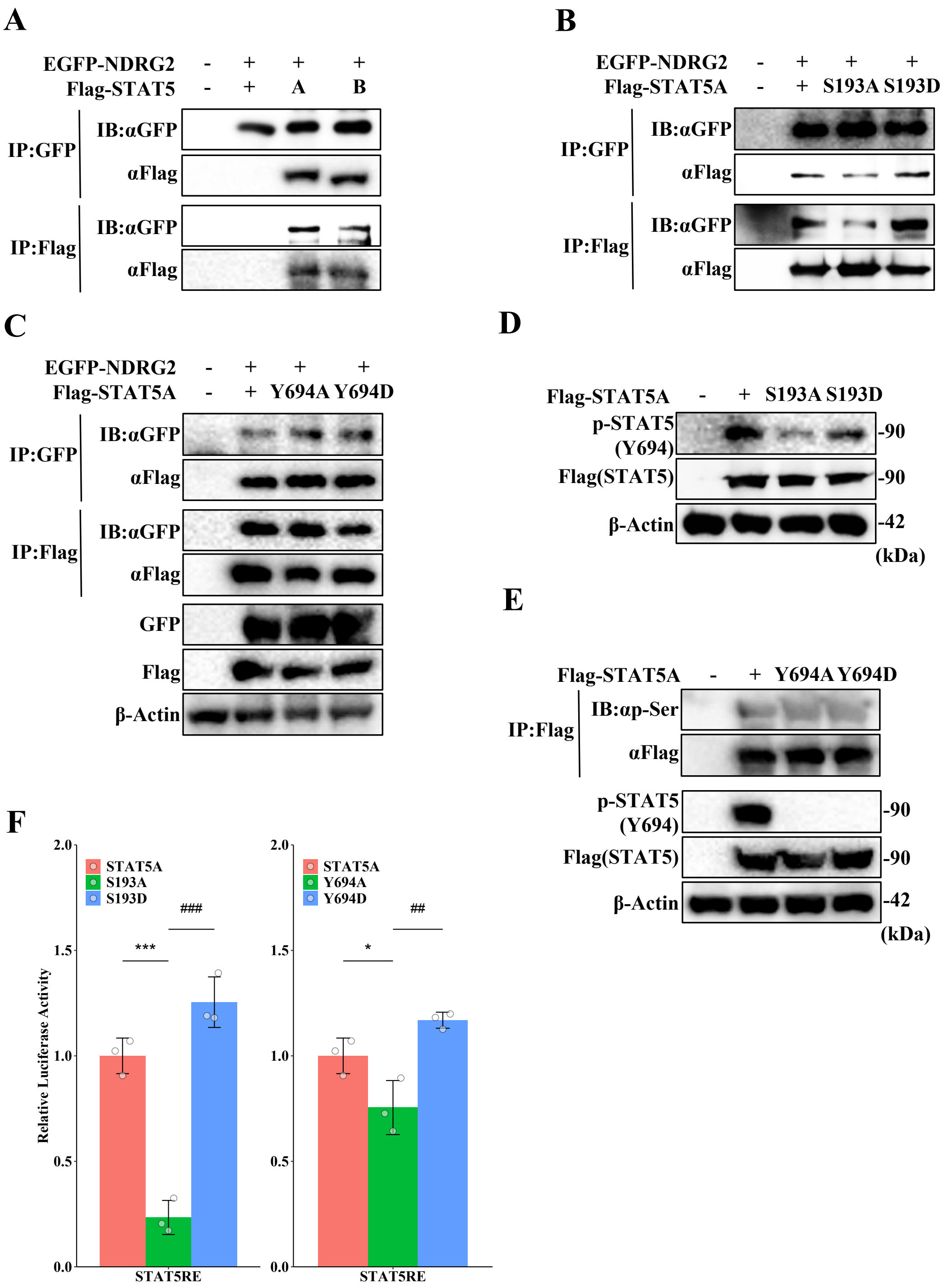

Figure S3.

A, 293T cells were co-transfected with EGFP-NDRG2 and Flag-STAT5 (STAT5A, and STAT5B), and immunoprecipitates were immunoblotted with anti-GFP or anti-Flag antibodies to

detect NDRG2 and STAT5. B, 293T cells were co-transfected with EGFP-NDRG2 and Flag-STAT5A mutants (WT, S193A, and S193D), and immunoprecipitates were immunoblotted with anti-GFP or anti-Flag antibodies to detect NDRG2 and STAT5A. C, 293T cells were co-transfected with EGFP-NDRG2 and Flag-STAT5A mutant (WT, Y694A, and Y694D). D, 293T cells were transfected with Flag-STAT5A mutant (WT, S193A, and S193D), and whole lysates were probed for the indicated antibodies. E, 293T cells were transfected with Flag-STAT5A mutant (WT, Y694A, and Y694D), and cell lysates were precipitated using anti-Flag antibody, and the precipitated proteins were immunoblotted with each specific antibody. F, 293T cells were transfected with Flag-STAT5A mutant, pSTAT5RE-Luc and pRL-TK plasmids, and subjected to luciferase reporter assays. The mean and SD are shown (n = 3); *$p < 0.05$, ***$p < 0.001$ versus STAT5A, ###$p < 0.001$ versus S193A, ##$p < 0.01$ versus Y694A.

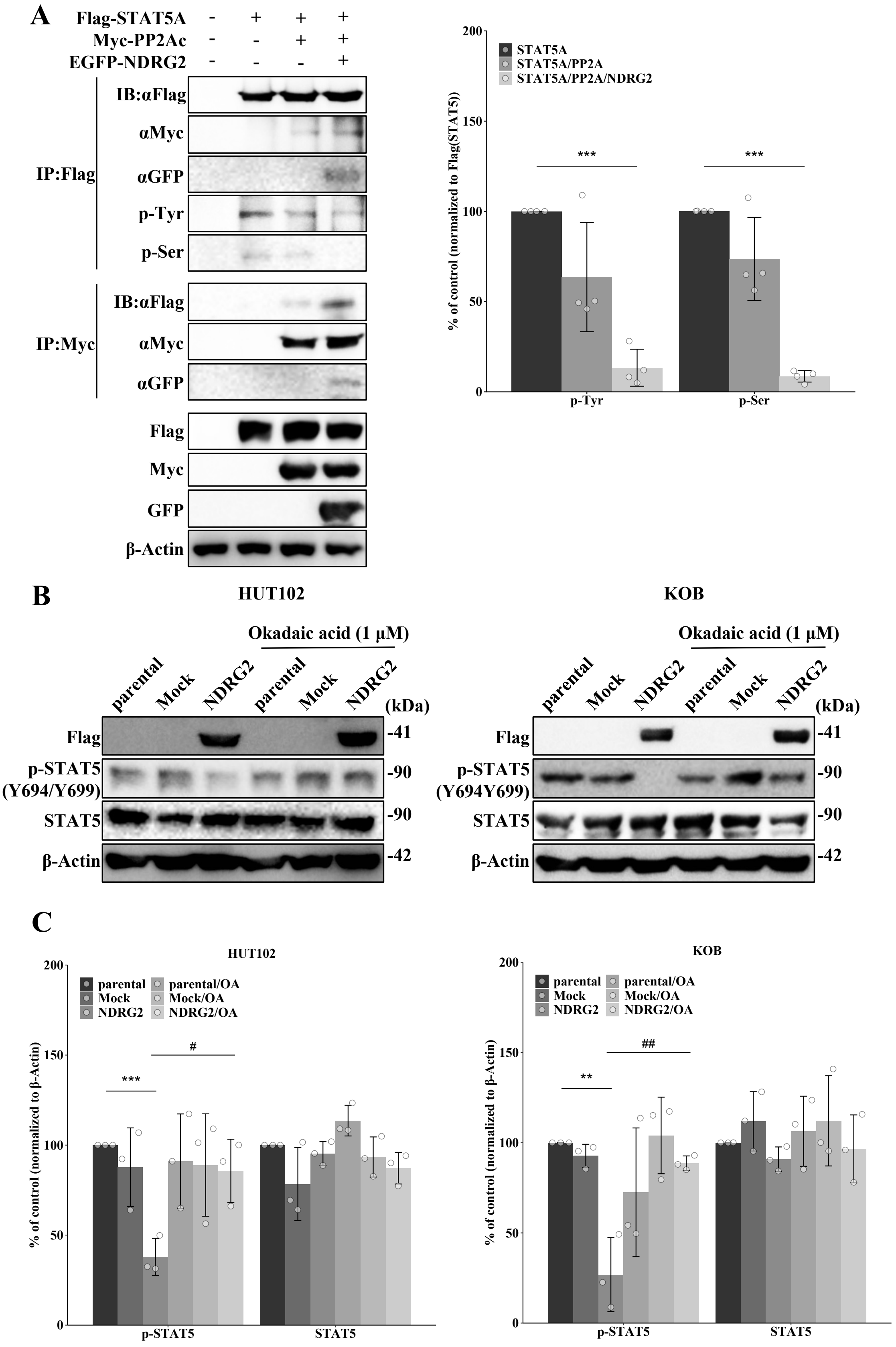
A
Flag-STAT5A - + + +
Myc-PP2Ac - - + +
EGFP-NDRG2 - - - +
IP:Flag
IB:αFlag
αMyc
αGFP
p-Tyr
p-Ser
IP:Myc
IB:αFlag
αMyc
αGFP
Flag
Myc
GFP
β-Actin
STAT5A
STAT5A/PP2A
STAT5A/PP2A/NDRG2
% of control (normalized to Flag(STAT5))
***
***
p-Tyr
p-Ser
B
HUT102
KOB
Okadaic acid (1 μM)
parental
Mock
NDRG2
(kDa)
Flag
p-STAT5 (Y694/Y699)
p-STAT5 (Y694Y699)
STAT5
β-Actin
-41
-90
-90
-42
C
HUT102
KOB
parental
Mock
NDRG2
parental/OA
Mock/OA
NDRG2/OA
% of control (normalized to β-Actin)
***
#
**
##
p-STAT5
STAT5

D

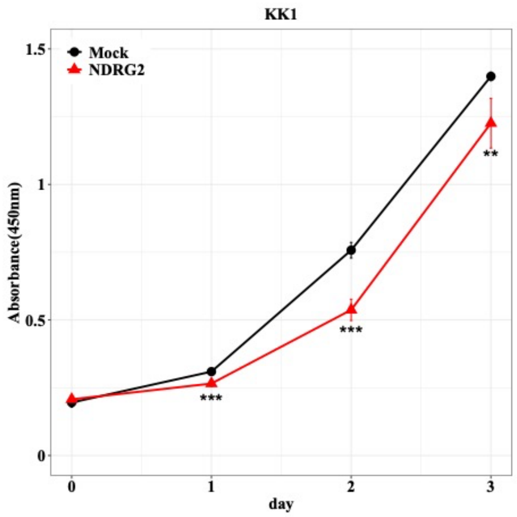

Figure S4.

A, Cell lysates of 293T cells transfected with Flag-STAT5A, Myc-PP2A, and EGFP-NDRG2 were immunoprecipitated with anti-Flag or anti-Myc antibodies, and immunoprecipitates were detected by western blotting using the indicated antibodies. Bar graphs show the quantification of the relative band intensity (p-Tyr and p-Ser) normalized to immunoprecipitated Flag(STAT5). Data are shown as mean and SD (n = 3); ***p < 0.001 versus STAT5A. B, HUT102 and KOB cells (parental, Mock, and NDRG2) were pretreated with or without Okadaic acid (1 μ M), and incubated for another 24 h, followed by western blot analysis using the indicated antibodies. C, Bar graphs show the quantification of relative band intensity normalized to β-actin. The mean and SD are shown (n = 3); **p < 0.01, ***p < 0.001 versus parental, and #p < 0.05, ##p < 0.01 versus NDRG2. D, Cell growth curves of KK1 cells (Mock and NDRG2) after 3 days. The mean and SD are shown (n = 4); **p < 0.01, ***p < 0.001 versus Mock.

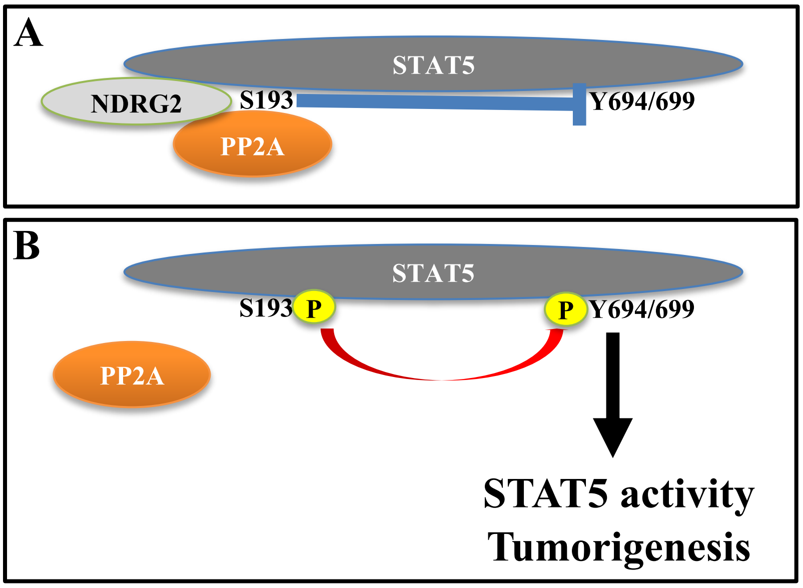

Figure S5.

A, NDRG2 directly binds to S193 site of STAT5B followed by the dephosphorylation via the recruitment of PP2A, resulting in the suppression of tyrosine phosphorylation in normal cells. B, The loss of NDRG2 caused the retention of S193 phosphorylation through the dissociation of PP2A followed by the enhancement of tyrosine phosphorylation and transcriptional activity.
